# Comparison of clinical, laboratory and radiological features in confirmed and unconfirmed COVID-19 patients

**DOI:** 10.2217/fmb-2021-0162

**Published:** 2021-11-23

**Authors:** Serkan Surme, Gulsah Tuncer, Betul Copur, Esra Zerdali, Inci Yilmaz Nakir, Meltem Yazla, Osman Faruk Bayramlar, Ahmet Buyukyazgan, Ayse Ruhkar Kurt Cinar, Hatice Balli, Yesim Kurekci, Filiz Pehlivanoglu, Gonul Sengoz

**Affiliations:** ^1^Department of Infectious Diseases & Clinical Microbiology, Haseki Training & Research Hospital, 34096, Istanbul, Turkey; ^2^Department of Medical Microbiology, Institute of Graduate Studies, Istanbul University-Cerrahpasa, 34098, Istanbul, Turkey; ^3^Department of Public Health, Bakirkoy District Health Directorate, 34140, Istanbul, Turkey

**Keywords:** close contact, complete blood count, COVID-19, diagnosis, platelet, SARS-CoV-2 PCR

## Abstract

**Background:** We aimed to compare the clinical, laboratory and radiological findings of confirmed COVID-19 and unconfirmed patients. **Methods:** This was a single-center, retrospective study. **Results:** Overall, 620 patients (338 confirmed COVID-19 and 282 unconfirmed) were included. Confirmed COVID‐19 patients had higher percentages of close contact with a confirmed or probable case. In univariate analysis, the presence of myalgia and dyspnea, decreased leukocyte, neutrophil and platelet counts were best predictors for SARS-CoV-2 RT-PCR positivity. Multivariate analyses revealed that only platelet count was an independent predictor for SARS-CoV-2 RT-PCR positivity. **Conclusion:** Routine complete blood count may be helpful for distinguishing COVID-19 from other respiratory illnesses at an early stage, while PCR testing is unique for the diagnosis of COVID-19.

COVID-19 has caused substantial morbidity and mortality [1]. Thus, testing for SARS-CoV-2 polymerase chain reaction (PCR) among suspected patients is decisive for the management including isolation, supportive care and treatment [2,3]. COVID-19 has also posed a challenge for healthcare resources. Although viral nucleic acid amplification real-time reverse-transcriptase PCR (RT-PCR) detection is widely accepted as the reference diagnostic method, some countries have faced problems such as a shortage of PCR kits and other types of equipment, certified laboratories and trained healthcare workers [4]. Additionally, many laboratories have faced a backlog of tests and delays in PCR results [5]. Moreover, there is a lack of hospital beds, personal protective equipment and isolation rooms [6,7], although it is recommended that hospitalized patients suspected of COVID-19 should not be cohorted with confirmed cases in the same wards [3]. Because of the shortage, easy and inexpensive predictive parameters for SARS-CoV-2 are needed for resource-limited settings.

Although the epidemiological characteristics and clinical features of COVID-19 patients have been well documented [1], there is still a challenge to discriminate COVID-19 from suspected patients. World Health Organization (WHO) and other healthcare authorities provide COVID-19 case definition, which comprises both epidemiological features and clinical characteristics as well as PCR testing [8]. However, these demographic and clinical characteristics are not specific for COVID-19. Previous studies have suggested that radiological examinations can help to diagnose COVID-19 [9]. Additionally, some laboratory parameters may be useful as a triage tool for patients with suspected COVID-19. Recent studies evaluate laboratory parameters as early predictors for COVID-19 diagnosis [10–14]. This encourages further investigation to understand the value of laboratory findings. However, none of hematological and biochemical tests studied is sufficient to be used as a standalone diagnostic test for COVID-19 in a recent Cochrane review study [10].

In this study, we aimed to compare epidemiological features, clinical characteristics, radiological findings and various laboratory parameters of molecularly confirmed COVID-19 and unconfirmed patients with negative SARS-CoV-2 PCR to provide useful and supplementary indicators for the diagnostic workup at an early stage. Additionally, we investigated the relationship between SARS-CoV-2 PCR positivity and mortality.

## Materials & methods

All hospitalized patients with suspected COVID-19 between 14 April 2020 and 14 May 2020 were included in this study. We retrospectively collected demographic characteristics, clinical features, laboratory parameters (leukocyte, neutrophil, lymphocyte, neutrophil/lymphocyte rate, monocyte, platelet, hemoglobin, hematocrit, glucose, urea, triglyceride, aspartate aminotransferase, alanine aminotransferase, lactate dehydrogenase, creatine kinase, albumin, sodium, potassium, calcium, ferritin, C-reactive protein, troponin, procalcitonin, fibrinogen and D-dimer), imaging findings, and outcomes from medical records via a data sheet form. All data were recorded at admission or within 24 h after hospitalization. Nasopharyngeal or oropharyngeal swabs were collected for SARS-CoV-2 RT-PCR. Samples tested negative were repeated after 48–72 h.

A suspected case was defined according to the guideline for COVID-19 of the General Directorate of Public Health division of the Turkish Ministry of Health [3]. The criteria are as follows;

- At least one of the signs and symptoms of fever or acute respiratory disease (cough and respiratory distress);
- Inability to explain the clinical manifestation with another cause/disease AND;
- A history of himself or his/her relative being abroad within 14 days before the onset of symptoms;

OR

- At least one of the signs and symptoms of fever or acute respiratory disease (cough and respiratory distress);
- Close contact with the confirmed COVID-19 case within 14 days before to the onset of symptoms;

OR

- At least one of the signs and symptoms of fever and severe acute respiratory infection (cough and respiratory distress);
- Presence of hospitalization requirement due to Severe Acute Respiratory Infections AND;
- Failure to explain the clinical manifestation with another cause/disease;

OR

- Cough or shortness of breath with a sudden start of fever and no nasal discharge.

A confirmed COVID-19 case was defined as a person diagnosed with a molecularly confirmed COVID-19 by SARS-CoV-2 RT-PCR among suspected patients. An unconfirmed case was defined as a person whose RT-PCR for SARS-CoV-2 results were negative in entirety.

All hospitalized patients were treated in isolation rooms. Sputum Gram stain and culture were obtained if a patient had symptoms or signs associated with bacterial infection (e.g., based on chest imaging, purulent sputum or acute deterioration). At least one set blood sample was obtained for the culture if a patient had a fever or increased acute phase reactants. Fever was defined as the body temperature measurement ≥38°C by non-contact infrared thermometry temperature measurement.

Owing to the shortage of urinary antigen tests for *Streptococcus pneumonia* and *Legionella* sp., serological tests for other atypical bacterial agents and multiplex PCR for viral respiratory tract pathogens, we did not perform these tests. However, if influenza was suspected, rapid influenza antigen test was obtained from hospitalized patients.

Mortality was defined as all-cause in-hospital death. The criteria for intensive care unit transfer in our hospital were the following parameters (at least one or more): 1) dyspnea and respiratory distress, 2) respiratory rate ≥30/minute, 3) oxygen saturation <90% or partial oxygen pressure <60 mmHg despite oxygen support (≥5 l/min) and 4) septic shock and/or multiple organ dysfunction.

Continuous variables were described as mean ± standard deviation (sd), while categorical variables were described as numbers and percentages. Chi-square and Fisher's exact tests were used to compare categorical variables. When the data of means were normally distributed, the Independent Sample T-test was used; otherwise, the Mann––Whitney U test was used to compare means. We performed univariate analysis, and all significant variables except parameters with high percentages (>25%) of missing values were included in multivariable logistic regressions. Odds ratio (OR) values with 95% CI were calculated. A p-value of <0.05 was considered statistically significant. Receiver operating characteristic (ROC) curve analyses were performed to determine the sensitivity, specificity, area under the curve (AUC), and optimal cut-off values of independent predictors. Statistical Package for Social Sciences (SPSS) 20.0 version was used for statistical analyses.

All procedures performed in studies involving human participants were in accordance with the ethical standards of the Declaration of Helsinki and the National Research Committee. This study was approved by the Ethics Committee of Haseki Training and Research Hospital (approval number: 358 date: 08/10/2020). Written informed consent was waived, given the retrospective nature of this study.

## Results

A total of 620 patients who had suspected COVID-19 were included. The mean age was 52.7 ± 15.1 years, and 333 (53.7%) patients were males. In-hospital death occurred in 45 (7.3%) patients. Seventy-five (12.1%) patients were admitted to the intensive care unit. Of the patients admitted to the intensive care unit, 67 (10.8%) required mechanical ventilation. Sixteen patients (2.6%) were healthcare workers. One hundred and fifty-six (25.6%) patients had a history of close contact with a confirmed or probable case. Three hundred and forty-nine (56.3%) patients had at least one comorbidity. The most common comorbidities were hypertension (n = 194, 31.3%) and diabetes mellitus (n = 171, 27.6%). No significant difference was observed between confirmed COVID-19 and unconfirmed patients in terms of comorbidities (all p-values >0.05). The most common symptoms at admission were cough (n = 475, 76.6%), fever (n = 338, 55.5%) and dyspnea (n = 251, 40.5%). Of 620 patients, 338 (54.5%) were confirmed COVID-19 cases, and 282 (45.5%) were unconfirmed patients. Confirmed COVID‐19 patients had higher percentages of history of close contact with a confirmed or probable case (30.8% vs 19.5%, p = 0.001), dyspnea (45.0% vs 36.7%, p = 0.035) and myalgia (14.8% vs 9.2%, p = 0.035) than unconfirmed patients. Mean body temperature was higher in confirmed cases than those with unconfirmed (36.9 ± 0.9 vs 36.7 ± 0.8, p = 0.005). Systolic (p = 0.946) and diastolic blood pressure (p = 0.168), respiratory rate (p = 0.372), heart rate (p = 0.664) and peripheral capillary oxygen saturation (p = 0.784) were not significantly different between groups. Table 1 & 2 show the demographic and clinical characteristics of patients with confirmed and unconfirmed COVID-19 at baseline.

**Table 1.** Demographic and clinical characteristics of confirmed COVID-19 and unconfirmed patients.

Parameters	In total, n (%)	SARS-CoV-2 RT-PCR		p-value
		Positive, n (%)	Negative, n (%)	
Patients (n)	620 (100)	338 (54.5)	282 (45.5)	
**Age (years)**				
Mean ± sd	52.7 ± 15.1	52.7 ± 15.0	52.7 ± 15.3	0.923
**Age group (years)**				
18–49	262 (42.3)	142 (42.0)	120 (42.6)	0.915
50–64	223 (36)	124 (36.7)	99 (35.1)	
65–74	80 (12.9)	43 (12.7)	37 (13.1)	
75–84	43 (6.9)	24 (7.1)	19 (6.7)	
>84	12 (1.9)	5 (1.5)	7 (2.5)	
**Age (years)**				
<65	485 (78.2)	266 (78.7)	219 (77.7)	0.755
≥65 years	135 (21.8)	72 (21.3)	63 (22.3)	
**Gender**				
Male	333 (53.7)	182 (53.8)	151 (53.5)	0.941
Female	287 (46.3)	156 (46.2)	131 (46.5)	
**Epidemiological history**				
Healthcare workers	16 (2.6)	11 (3.3)	5 (1.8)	0.247
Close contact with a confirmed or probable case	159 (25.6)	104 (30.8)	55 (19.5)	**0.001**
Underlying diseases	349 (56.3)	188 (55.6)	161 (57.1)	0.713
– COPD	25 (4.0)	11 (3.3)	14 (5.0)	0.281
– Diabetes mellitus	171 (27.6)	98 (29.0)	73 (25.9)	0.389
– Hypertension	194 (31.3)	107 (31.7)	87 (30.9)	0.829
– Congestive heart failure	12 (1.9)	7 (2.1)	5 (1.8)	0.789
– Chronic artery disease	62 (10.0)	37 (10.9)	25 (8.9)	0.390
– Chronic renal failure	33 (5.3)	15 (4.4)	18 (6.4)	0.283
– Malignancy	10 (1.6)	8 (2.4)	2 (0.7)	0.121
**Clinical outcomes**				
Invasive ventilation	67 (10.8)	44 (13.0)	23 (8.2)	0.052
ICU admission	75 (12.1)	49 (14.5)	26 (9.2)	**0.045**
Death	45 (7.3)	31 (9.2)	14 (5.0)	**0.044**

The p-values <0.05 in bold are statistically significant.

COPD: Chronic obstructive pulmonary disease; ICU: Intensive care unit.

**Table 2.** Symptoms and vital signs of confirmed COVID-19 and unconfirmed patients.

Symptoms	In total, n (%)	SARS-CoV-2 RT-PCR		p-value
		Positive, n (%)	Negative, n (%)	
Fever	344 (55.5)	196 (58.0)	148 (52.5)	0.170
Cough	475 (76.6)	267 (79.0)	208 (73.8)	0.125
Dyspnea	251 (40.5)	127 (45.0)	124 (36.7)	**0.035**
Rhinorrhea	7 (1.1)	1 (0.3)	6 (2.1)	0.051
Wheezing	4 (0.6)	2 (0.6)	2 (0.7)	1.000
Chest pain	19 (3.1)	10 (3.0)	9 (3.2)	0.867
Myalgia	76 (12.3)	50 (14.8)	26 (9.2)	**0.035**
Arthralgia	21 (3.4)	14 (4.1)	7 (2.5)	0.255
Fatigue	218 (35.2)	129 (38.2)	89 (31.6)	0.086
Sore throat	53 (8.5)	23 (6.8)	30 (10.6)	0.089
Abdominal pain	11 (1.8)	8 (2.4)	3 (1.1)	0.221
Nausea	40 (6.5)	23 (6.8)	17 (6.0)	0.695
Vomiting	20 (3.2)	12 (3.6)	8 (2.8)	0.617
Diarrhea	25 (4.0)	10 (3.0)	15 (5.3)	0.137
**Vital signs**				
Systolic blood pressure	118 ± 13	121 ± 13	114 ± 12	0.946
Diastolic blood pressure	73 ± 9	74 ± 9	72 ± 9	0.168
Body temperature	36.8 ± 0.8	36.9 ± 0.9	36.7 ± 0.8	**0.006**
Respiratory rate/minute	21.5 ± 4.2	21.7 ± 4.3	21.3 ± 4.1	0.372
Heart rate/minute	87.9 ± 13.3	88.1 ± 13.5	87.6 ± 13.1	0.664
SpO2	92.9 ± 5.6	93.0 ± 5.9	92.9 ± 5.1	0.784

The p-values <0.05 in bold are statistically significant.

SpO2: Peripheral capillary oxygen saturation.

Regarding microbiological evaluation, bacterial microorganisms were isolated in sputum samples from 18 patients (6.4%) of 282 unconfirmed patients. Only four patients (1.4%) had bacteremia. An influenza antigen test was positive in four patients (1.4%).

On admission, 25 (7.4%) patients did not have chest computed tomography (CT) imaging abnormalities in confirmed patients and seven (2.5%) in unconfirmed patients. Regarding chest CT findings, 94.5% of confirmed patients (n = 296/313) and 94.1% (n = 259/275) of unconfirmed patients had bilateral lung involvement. In chest CT, the most common finding was ground-glass opacity (n = 534/601, 88.9%), following consolidation (n = 183/601, 30.4%), and small patch (n = 136/601, 22.6%). Pulmonary nodules were less frequent in confirmed patients than in unconfirmed patients (6.1% vs 10.5%, p = 0.049), while other radiological findings did not significantly differ between groups. The radiological findings on admission are summarized in Table 3.

**Table 3.** Radiological findings of confirmed COVID-19 and unconfirmed patients.

Parameters	In total, n (%)	SARS-CoV-2 RT-PCR		p-value
		Positive, n (%)	Negative, n (%)	
**Chest radiography findings (n = 353)**				
Unilateral	38 (10.8)	19 (10.2)	19 (11.4)	0.697
Bilateral	315 (89.2)	168 (89.8)	147 (88.6)	
**Chest CT findings (n = 601)**				
Unilateral	33 (5.5)	17 (5.4)	16 (5.8)	0.847
Bilateral	556 (92.5)	296 (94.3)	259 (94.2)	
Small patch	136 (22.6)	75 (24.0)	61 (22.2)	0.583
Ground glass	534 (88.9)	285 (90.8)	249 (90.5)	0.685
Consolidation	183 (30.4)	101 (32.3)	82 (29.8)	0.508
Air bronchogram	26 (4.3)	14 (4.5)	12 (4.4)	0.943
Interlobular septal thickening	52 (8.6)	28 (8.9)	24 (8.7)	0.917
Pulmonary nodules	48 (8.0)	19 (6.1)	29 (10.5)	**0.049**
Pathological lymph node	9 (1.0)	4 (1.3)	5 (1.8)	0.740
**Pleural fluid (n = 29)**				
Unilateral	8 (27.5)	5 (38.5)	3 (18.8)	0.406
Bilateral	21 (72.5)	8 (61.5)	13 (81.2)	

The p-values <0.05 in bold are statistically significant.

CT: Computed tomography.

The mean counts of leukocytes, neutrophils and platelets in confirmed COVID‐19 patients were in the normal range. Leukocyte count (6085 ± 2386 vs 6940 ± 3034, p < 0.001), neutrophil count (4076 ± 2017 vs 4830 ± 2709, p < 0.001) platelets (192 10^3^/μl vs 220 10^3^/μl, p = 0.001), and lactate dehydrogenase (287.2 ± 107.2 vs 309.9 ± 126.8 U/l, p = 0.022) were lower in confirmed patients than in unconfirmed patients. Lymphocyte count (p = 0.123), neutrophil to lymphocyte ratio (p = 0.112), monocyte count (p = 0.518), hemoglobin (p = 0.197), hematocrit (p = 0.767), glucose (p = 0.555), urea (p = 0.785), triglyceride (p = 0.757), aspartate aminotransferase (p = 0.761), alanine aminotransferase (p = 0.795), creatine kinase (p = 0.848), albumin (p = 0.083), sodium (p = 0.076), potassium (p = 0.816), calcium (p = 0.637), procalcitonin (p = 0.693), ferritin (p = 0.629), troponin (p = 0.748), C-reactive protein (p = 0.278), fibrinogen (p = 0.350), and D-dimer (p = 0.077) did not significantly differ between groups (all p > 0.05). The laboratory findings on admission are shown in Table 4.

**Table 4.** Laboratory findings of confirmed COVID-19 and unconfirmed patients.

Parameters	In total	SARS-CoV-2 RT-PCR		p-value
		Positive	Negative	
Leukocyte (μl)	6476 ± 2732	6085 ± 2386	6940 ± 3034	**<0.001**
Neutrophil (μl)	4421 ± 2387	4076 ± 2017	4830 ± 2709	**<0.001**
Lymphocyte (μl)	1439 ± 646	1409 ± 638	1475 ± 654	0.123
Neutrophil lymphocyte ratio	3.68 ± 2.85	3.48 ± 2.59	3.92 ± 3.12	0.112
Monocyte (μl)	722.0 ± 1265	682 ± 1280	769 ± 1247	0.518
Monocyte (%)	8.76 ± 3.60	8.92 ± 3.80	8.57 ± 3.34	0.577
Platelet (10^3^ μl)	205 ± 75	192 ± 68	220 ± 81	**0.001**
Hemoglobin (g/dl)	13.0 ± 1.7	13.0 ± 1.7	12.9 ± 1.6	0.197
Hematocrit (%)	38.8 ± 4.8	38.9 ± 4.9	38.8 ± 4.7	0.767
Glucose (mg/dl)	139.0 ± 68.0	138.3 ± 62.9	139.7 ± 73.6	0.555
Urea (mg/dl)	39.5 ± 165.5	32.5 ± 23.1	47.9 ± 243.5	0.785
Creatinine (mg/dl)	0.99 ± 1.29	0.91 ± 0.74	1.10 ± 1.72	0.776
Triglyceride (mg/dl)	124.4 ± 50.5	125.6 ± 54.9	123.0 ± 45.1	0.757
AST (IU/l)	37.7 ± 27.3	37.5 ± 25.9	37.9 ± 29.0	0.761
ALT (IU/l)	29.8 ± 31.6	30.4 ± 35.7	29.2 ± 25.8	0.795
LDH (U/l)	297.9 ± 117.3	287.2 ± 107.2	309.9 ± 126.8	**0.022**
CK (IU/l)	182.3 ± 238.6	194.5 ± 282.2	167.7 ± 172.6	0.848
Albumin (g/l)	36.8 ± 4.4	37.2 ± 4.2	36.3 ± 4.6	0.083
Sodium (mmol/l)	137 ± 3	136.7 ± 3	137 ± 4	0.076
Potassium (mmol/l)	4.22 ± 2.20	4.30 ± 2.95	4.13 ± 0.56	0.816
Calcium (mmol/l)	8.80 ± 0.77	8.79 ± 0.77	8.80 ± 0.78	0.637
Ferritin (ng/ml)	252.5 ± 304.0	244.3 ± 235.1	260.8 ± 362.2	0.629
CRP (mg/l)	61.0 ± 61.3	58.1 ± 57.4	64.5 ± 65.6	0.278
Troponin (mg/dl)	35.3 ± 209.0	51.0 ± 286.4	19.1 ± 64.5	0.748
Procalcitonin (μg/l)	0.18 ± 0.49	0.18 ± 0.54	0.17 ± 0.43	0.693
Fibrinogen (mg/dl)	476.0 ± 122.5	471.7 ± 122.7	480.6 ± 122.7	0.350
D-dimer (μg/l)	1.59 ± 4.52	1.15 ± 3.16	2.07 ± 5.62	0.077

The p-values <0.05 in bold are statistically significant.

ALT: Alanine aminotransferase; AST: Aspartate aminotransferase; CK: Creatine kinase; CRP: C-reactive protein; LDH: Lactate dehydrogenase.

In univariate analysis, presence of myalgia (p = 0.037, OR = 1.709 CI = 1.034–2.827) and dyspnea (p = 0.035, OR = 1.414 CI = 1.024–1.952), decreased leukocyte (p = 0.008, OR = 1.564 CI = 1.127–2.172), neutrophil (p = 0.008, OR = 1.564 CI = 1.124–2.177) and platelet counts (p = 0.001, OR = 1.735 CI = 1.255–2.398) were best predictors for SARS-CoV-2 RT-PCR positivity among patients with suspected COVID-19. Multivariate analyses revealed that only platelet count was an independent predictor for SARS-CoV-2 RT-PCR positivity (Table 5). ROC curve analysis suggested a cut-off value for platelet count as 181.5 (10^3^/μl, larger values indicate SARS-CoV-2 RT-PCR positivity) for the diagnosis of COVID-19 with the sensitivity and specificity of 51.8% and 37.1%, respectively. The sensitivity increased up to 74.9% at a cut-off value of 150 10^3^/μl (AUC = 0.601, p < 0.001).

**Table 5.** Univariate and multivariate analyses of factors predicting SARS-CoV-2 RT-PCR positivity.

Logistic regression	Univariate			Multivariate		
	OR	CI	p-value	OR	CI	p-value
Dyspnea	1.414	1.024–1.952	**0.035**	1.342	0.884–1.884	0.084
Myalgia	1.709	1.034–2.827	**0.037**	1.446	0.853–2.452	0.171
Body temperature	1.254	0.914–1.725	0.170	–	–	–
Pulmonary nodules	1.816	0.994–3.316	0.052	–	–	–
Leukocyte count	1.564	1.127–2.172	**0.008**	–	–	–
Neutrophil count	1.564	1.124–2.177	**0.008**	1.263	0.884–1.804	0.200
Platelet count/ul	1.735	1.255–2.398	**0.001**	1.612	1.140–2.280	**0.007**
LDH	1.388	0.968–1.989	0.074	–	–	–

The p-values <0.05 in bold are statistically significant.

LDH: Lactate dehydrogenase.

Additionally, confirmed COVID-19 patients had significantly poorer outcomes compared to unconfirmed patients. Intensive care unit admission (14.5% vs 9.2%, p = 0.045) and in-hospital death (9.2% vs 5.0%, p = 0.044) were more common in confirmed cases than those with unconfirmed. However, there was no significant difference between confirmed and unconfirmed patients regarding mechanical ventilation (13.0% vs 8.2%, p = 0.052). In univariate analysis, age (p < 0.001, OR = 2.439 CI = 1.460–4.082), dyspnea (p < 0.001, OR = 3.204, CI = 1.686–6.089), presence of any comorbidity (p = 0.019, OR = 2.254 CI = 1.141–4.453), hypertension (p = 0.023, OR = 2.033, CI = 1.102–3.750), chronic artery disease (p = 0.006, OR = 2.874 CI = 1.346–6.135), chronic renal failure (p < 0.001, OR = 5.504 CI = 2.160–11.608), peripheral capillary oxygen saturation (p < 0.001, OR = 1.164 CI = 1.111–1.128), body temperature (p = 0.035, OR = 0.707 CI = 0.511–0.977), neutrophil to lymphocyte ratio (p < 0.001, OR = 0.840 CI = 0.779–0.906), creatinine (p < 0.001, OR = 0.704, CI = 0.585–0.848), C-reactive protein (p < 0.001, OR = 0.987 CI = 0.983–0.991), sodium (p = 0.001, OR = 1.150, CI = 1.055–1.253), consolidation in chest CT (p = 0.002, OR = 2.223, CI = 1.352–3.656), air bronchogram in chest CT (p = 0.021, OR = 3.371, CI = 1.204–9.436), and SARS-CoV-2 RT-PCR positivity (p = 0.048, OR = 1.933, CI = 1.007–9.710) were associated with in-hospital death. Multivariate analysis revealed that SARS-CoV-2 RT-PCR positivity was an independent predictor for increased in-hospital death (p = 0.034, OR = 2.66 CI = 1.074–6.324), when we adjusted with potential confounding factors in the univariate analysis including age, dyspnea, comorbidities (hypertension, chronic artery disease and chronic renal failure), vital signs (peripheral capillary oxygen saturation, body temperature), laboratory parameters (neutrophil to lymphocyte ratio, creatinine, C-reactive protein and sodium), radiological findings (consolidation in chest CT and air bronchogram in chest CT; Table 6).

**Table 6.** Univariate and multivariate analysis of factors predicting mortality.

Logistic regression	Univariate			Multivariate		
	OR	CI	p-value	OR	CI	p-value
Age	2.439	1.460–4.082	**<0.001**	4.151	1.825–9.439	**0.001**
Dyspnea	3.204	1.686–6.089	**<0.001**	1.877	0.779–4.552	0.611
Any comorbidity	2.254	1.141–4.453	**0.019**	0.881	0.251–3.089	0.843
Hypertension	2.033	1.102–3.750	**0.023**	1.644	0.538–5.025	0.384
Chronic artery disease	2.874	1.346–6.135	**0.006**	0.900	0.280–2.892	0.859
Chronic renal failure	5.504	2.160–11.608	**<0.001**	1.704	0.290–9.998	0.555
SpO2	1.164	1.111–1.128	**<0.001**	1.091	1.040–1.144	**<0.001**
Body temperature	0.707	0.511–0.977	**0.035**	0.851	0.489–1.456	0.556
NLR	0.840	0.779–0.906	**<0.001**	0.952	0.835–1.086	0.468
Creatinine	0.704	0.585–0.848	**<0.001**	0.764	0.612–0.955	**0.018**
CRP	0.987	0.983–0.991	**<0.001**	0.988	0.982–0.993	**<0.001**
Sodium	1.150	1.055–1.253	**0.001**	1.020	0.901–1.155	0.760
Consolidation in chest CT	2.223	1.352–3.656	**0.002**	3.165	1.391–7.202	**0.006**
Air bronchogram in chest CT	3.371	1.204–9.436	**0.021**	2.441	0.546–10.902	0.243
SARS-CoV-2 RT-PCR	1.933	1.007–9.710	**0.048**	2.606	1.074–6.324	**0.034**

The p-values <0.05 in bold are statistically significant.

CRP: C-reactive protein; CT: Computed tomography; NLR: Neutrophil lymphocyte ratio; PCR: Polymerase chain reaction; SpO2: Peripheral capillary oxygen saturation.

## Discussion

As COVID-19 has caused serious and fatal outcomes, prompt and accurate diagnosis is vital [1]. In this study, we present a comprehensive analysis of the clinical characteristics, laboratory test results, radiological findings and outcomes of 338 confirmed COVID‐19 patients with those of 282 unconfirmed patients in a setting, which hospitalizes suspected COVID-19 patients who have comparable chest CT findings. In univariate analysis, the presence of myalgia and dyspnea, decreased leukocyte, neutrophil and platelet counts were best predictors for SARS-CoV-2 RT-PCR positivity among patients with suspected COVID-19. Among these parameters, only platelet count is an independent predictor for SARS-CoV-2 RT-PCR positivity. However, ROC curve analysis revealed that platelet counts had low accuracy for the diagnosis (AUC = 0.601). In addition, we found that SARS-CoV-2 RT-PCR positivity was an independent predictor for in-hospital death and associated with about 3-fold increased risk, consistent with other studies [15,16].

In the present study, the baseline characteristics of COVID-19, such as common comorbidities, symptoms, laboratory and radiological findings were consistent with the previous studies on COVID-19 in Turkey [11,17]. Confirmed COVID-19 patients were not more likely to have any of the underlying diseases than unconfirmed patients. Besides epidemiological exposure history, dyspnea and myalgia appeared more frequently in confirmed COVID‐19 patients than in unconfirmed patients. In addition, body temperature on admission was slightly higher in confirmed patients. In this study, we verified that fever, cough and dyspnea were the main symptoms of both confirmed and unconfirmed patients. In addition, most symptoms presented in confirmed COVID‐19 patients and unconfirmed patients with similar percentages. As a result, symptoms had limited effects on the differentiation of COVID‐19 from unconfirmed patients, although dyspnea and myalgia were more common in confirmed COVID‐19 patients. Similarly, Li *et al.* reported that confirmed patients were more likely to present with dyspnea (79% vs 61%, p = 0.028) and myalgia (57% vs 34%, p = 0.017), while other symptoms did not significantly differ between groups [18]. In the study of Zhou *et al.*, fever, dyspnea, fatigue, chest distress were more common in confirmed COVID-19. Compared to confirmed COVID-19, patients with SARS-CoV-2 negative community-acquired pneumonia had more likely chronic obstructive pulmonary disease (COPD) and malignancy. However, SARS-CoV-2 negative patients were mainly included from a respiratory department treating especially patients with COPD and pulmonary tumors. Additionally, they revealed that confirmed COVID-19 patients had more likely leukopenia, hypoalbuminemia, increased C-reactive protein and lactate dehydrogenase levels. However, no difference was observed in platelet count between two groups [19]. In the study of Saegerman *et al.*, fever (OR = 3.66) and dry cough (OR = 1.71) were the two most relevant symptoms of COVID-19. Chest pain (OR = 0.73) and sore throat (OR = 0.73) were significantly less associated with the confirmed COVID-19 [20]. Feng *et al.* reported that only myalgia was significantly higher in confirmed COVID-19 than in unconfirmed patients (85.7% vs 26.3%, p = 0.021) [12]. Sun *et al.* reported that epidemiological exposure and body temperature but not comorbidities were higher in confirmed COVID-19 than unconfirmed patients, in consistent with our study. However, no difference was found in other vital signs or the presence of cough and dyspnea [21]. In the study of Sun *et al.*, they also found that leukocyte, neutrophil, lymphocyte, platelet counts were higher in confirmed COVID-19.

Serum biomarkers are often used as routines in emergency departments, and they are mostly inexpensive. Some laboratory parameters are significantly increased or decreased in confirmed COVID-19 when compared with unconfirmed patients [11–14,17–21]. In the study conducted by Şan *et al.*, leukocyte (5150 vs 7100, p < 0.001), neutrophil (3300 vs 4660, p < 0.001), and platelet counts (209 10^3^/μl vs 249 10^3^/μl, p < 0.001) were lower in confirmed COVID-19 [11]. Also, they found additional diagnostic hematological parameters such as lymphocyte count, monocyte count, platelecrit, fibrinogen concentration and D-dimer. Previous studies demonstrated that lymphopenia could be one of the typical characteristics of COVID-19 and related to COVID-19 severity. However, lymphocyte count did not significantly differ between groups of confirmed COVID-19 and unconfirmed patients in our study. Our study confirmed several studies which reported lower platelets in confirmed COVID-19 patients [13,14,22]. However, these studies did not demonstrate platelet count as an independent predictor for confirmed COVID-19. In the study of Feng *et al.*, there were three groups including suspected COVID-19, confirmed COVID-19 and unsuspected patients. Leukocyte, neutrophil, and lymphocyte counts were lower in confirmed COVID-19 than non-confirmed, while platelet and lymphocyte counts were lower in suspected COVID-19 than unsuspected [12]. In another study, COVID-19 patients had higher AST levels, and lower leukocyte, neutrophil, lymphocyte, eosinophil and monocyte counts [13]. The decreased levels in complete blood count could have resulted from the immunosuppression and systemic inflammatory response during the disease development and progression [23]. Zhou *et al.* revealed that confirmed COVID-19 patients had more likely leukopenia, hypoalbuminemia, increased C-reactive protein and lactate dehydrogenase levels [19]. As a result, laboratory parameters are not specific. This may be because of certain effects of COVID-19 including pulmonary, cardiac, renal and hepatic complications [24]. This reflects the complexity of COVID-19. Thus, a single specific biomarker has not been used as a diagnostic test.

Radiological examinations have been used to support clinical decision for COVID-19, although chest CT findings are variable [25]. Air-space consolidation in chest radiography is mostly bilateral [26,27]. In our study, chest CT images demonstrated mostly bilateral ground-glass opacities, in consistent with other studies [28,29]. However, the specificity of chest CT was low due to false positive findings. In addition, even radiological examinations cannot completely exclude COVID-19 [30,31] and they alone are not recommended for COVID-19 diagnosis [32]. In the present study, we did not find any substantial difference in chest CT findings. Similarly, Angelis *et al.* reported that there was no significant difference in ground-glass opacity, pleural thickening, fibrotic streaks, air bronchogram, and bronchus distortion between confirmed COVID-19 and unconfirmed patients [33]. In contrast, Miao *et al.* demonstrated that ground-glass opacity, crazy-paving pattern, air bronchogram, and pleural thickening were seen more frequent in confirmed COVID-19 patients than unconfirmed patients [34].

In the study of Lascarrou *et al*., four parameters were independent predictors for false negative first SARS-CoV-2 RT-PCR test as follows headache (OR = 0.07 CI = 0.01–0.49, p  =  0.007) and fatigue/malaise (OR = 0.16, CI = 0.03–0.81, p  =  0.027), platelets >207 10^3^ mm^3^ (OR = 3.81 CI = 1.10–13.16, p  =  0.034) and C-reactive protein >79.8 mg/l (OR = 4.00, CI = 1.21–13.19, p  =  0.023) [35]. In addition, they did not find any association between the false negative SARS-CoV-2 RT-PCR test and mortality (13.9% vs 15.2% p = 0.80). In the present study, we found an increased mortality in confirmed COVID-19 patients than in repeated SARS-CoV-2 RT-PCR negative unconfirmed patients (9.2% vs 5.0%, p = 0.044). However, a part of unconfirmed SARS-CoV-2 negative patients might still have COVID-19 even if repeated PCR tests were performed in our study. Antigen tests could be used as an auxiliary diagnosis tool for the discrimination of COVID-19 to mitigate false-negative diagnosis in the early stages of infection [36,37], but the sensitivity of antigen tests is lower compared to nucleic acid amplification tests. Therefore, negative results could be possibly false negative [38–40]. Antibody testing may be used as a rapid serological detection for the COVID-19 diagnosis. However, owing to the low sensitivity antibody testing is not preferred for acute COVID-19 infection detection [41].

The differential microbiological diagnosis of patients with respiratory tract infections is of importance to implement antimicrobial stewardship and to improve patient outcomes. In addition, microbiological evaluation is vital for defining the local epidemiology [42,43]. The opportunities of rapid molecular respiratory tests for microbiologic evaluation have increased [44,45]. While the predictive value of multiplex PCR tests is limited due to colonizations of some bacterial or viral pathogens, implementation of these tests would improve the accurate microbiological diagnosis. Nevertheless, availability and affordability are limited especially in resource-poor settings. Moreover, negative results obtained by nasopharyngeal or oropharyngeal swabs cannot exclude viral infections, and lower respiratory tract sampling (sputum and bronchoalveolar lavage fluid) may be required [46–48].

In the study of Hagman *et al.*, SARS-CoV-2 RT-PCR positive patients had poorer outcomes compared with unconfirmed PCR negative patients [15]. In their study, 15 out of 61 patients (24.6%) had all-cause mortality among confirmed patients, while only three out of 106 unconfirmed patients (2.8%) died. They found that age, underlying diseases, and C-reactive protein and SARS-CoV-2 RT-PCR positivity were associated with critical illnesses or death in the univariate analysis. Only SARS-CoV-2 RT-PCR positivity and age were independently associated with increased poor outcomes (critical illnesses or death). PCR positive confirmed patients had a seven-fold increased risk for critical illnesses and an eight-fold increased risk for mortality in multivariate regression analysis. Similarly, Prebensen *et al.* reported that SARS-CoV-2 RT-PCR positivity in plasma was higher in patients who were died and/or transferred to the intensive care unit compared to those who were not. Additionally, they detected higher plasma SARS-CoV-2 RNA levels in patients with poor outcomes [16]. SARS-CoV-2 triggers the immune response and cause dysregulation of the inflammatory mediators. This results in a cytokine storm which is proposed as a significant key element in the pathogenesis of severe illness [49]. This cytokine storm which could be due to leakage from tissues damaged may cause mortality in patients with COVID-19. In our study, we did not measure plasma SARS-CoV-2 RNA level, but nasopharyngeal or oropharyngeal PCR positivity was associated with about a three-fold increased risk of mortality. In contrast, in an Italian cohort of hospitalized for suspected COVID-19, there was no significant difference between confirmed and unconfirmed patients in terms of mortality [33]. They found that a high level of hemoglobin and a low level of leukocyte count were independently associated with confirmed COVID-19 diagnosis.

In conclusion, epidemiological exposure, dyspnea and myalgia are important features discriminating confirmed COVID‐19 from unconfirmed patients, and a positive RT-PCR testing are associated with poor outcomes. Additionally, COVID‐19 patients more frequently have slight changes in laboratory parameters such as leukocyte, neutrophil and platelet counts than unconfirmed patients.

This study had several strengths. First, different types of variables such as multiple comorbidities, symptoms, vital signs, laboratory and radiological parameters were included in the multivariate regression analysis. Second, we had relatively a large sample size. Third, it is noteworthy that the SARS-CoV-2 RT-PCR testing in our hospital was not restricted. Our study had also several limitations. First, it was retrospectively conducted in a single center. Second, SARS-CoV-2 antigen detection was not performed. PCR testing of swab samples might not be sufficiently accurate to determine COVID-19, although repeated tests were obtained from patients with negative SARS-CoV-2 RT-PCR. However, even consecutive non-reactive results do not rule out the possibility of COVID-19 [2]. Thus, we emphasize that clinicians should be aware of this possibility. In addition, we did not perform multiplex RT-PCR to determine infections with other respiratory pathogens such as influenza virus, parainfluenza virus, rhinovirus, respiratory syncytial virus, adenovirus and other coronaviruses or causative bacterial agents in unconfirmed patients. In our study, we did not identify any other causative agents of most unconfirmed patients. Even isolation of bacterial agents or detection of other viral respiratory pathogens does not rule out COVID-19 [50,51]. Finally, we did not perform longitudinal evaluations of laboratory parameters.

## Conclusion

Our data justify that there are significant differences in epidemiological and clinical characteristics, laboratory parameters and outcomes between confirmed COVID-19 and unconfirmed patients. Some hematological parameters but not biochemical markers of patients with COVID-19 were significantly different from unconfirmed patients. Routine complete blood count may also be helpful for distinguishing COVID-19 from other respiratory illnesses at an early stage, while PCR testing is unique for the diagnosis of COVID-19. Additionally, clinicians should consider PCR positivity as a predictor of mortality among hospitalized patients with suspected COVID-19.

**Summary points**

- Although the epidemiological characteristics and clinical features of COVID-19 patients have been well documented, there is still a challenge to discriminate COVID-19 patients from suspected patients.
- In the present study, confirmed COVID‐19 patients had higher percentages of close contact with a confirmed or probable case.
- In univariate analysis, the presence of myalgia and dyspnea, decreased leukocyte, neutrophil and platelet counts were best predictors for SARS-CoV-2 RT-PCR positivity.
- Multivariate analyses revealed that only platelet count was an independent predictor for SARS-CoV-2 RT-PCR positivity.
- SARS-CoV-2 RT-PCR positivity was an independent predictor for in-hospital death and was associated with about three-fold increased risk.
- This study suggests that routine complete blood count may be helpful for distinguishing COVID-19 from other respiratory illnesses at an early stage, while PCR testing is unique for the diagnosis of COVID-19.
- Clinicians should consider PCR positivity as a predictor of mortality among hospitalized patients with suspected COVID-19.
